# Comprehensive Evaluation of Polyaniline-Doped Lignosulfonate in Adsorbing Dye and Heavy Metal Ions

**DOI:** 10.3390/ijms25010133

**Published:** 2023-12-21

**Authors:** Wenjuan Wu, Penghui Li, Mingkang Wang, Huijun Liu, Xiufu Zhao, Caiwen Wu, Jianpeng Ren

**Affiliations:** 1 Jiangsu Co-Innovation Center of Efficient Processing and Utilization of Forest Resources, Nanjing Forestry University, Nanjing 210037, China; liph@njfu.edu.cn (P.L.);; 2 College of Light Industry and Food Engineering, Nanjing Forestry University, Nanjing 210037, Chinajpeng@njfu.edu.cn (J.R.); 3 School of Chemistry and Chemical Engineering, Southeast University, Nanjing 211189, China; 220222927@seu.edu.cn

**Keywords:** lignosulfonate, PANI, adsorption, dyes, malachite green

## Abstract

Lignosulfonate/polyaniline (LS/PANI) nanocomposite adsorbent materials were prepared by the chemical polymerization of lignosulfonate with an aniline monomer as a dopant and structure-directing agent, and the adsorption behavior of dyes as well as heavy metal ions was investigated. LS/PANI composites were used as dye adsorbents for the removal of different cationic dyes (malachite green, methylene blue, and crystal violet). The adsorption behavior of LS/PANI composites as dye adsorbents for malachite green was investigated by examining the effects of the adsorbent dosage, solution pH, initial concentration of dye, adsorption time, and temperature on the adsorption properties of this dye. The following conclusions were obtained. The optimum adsorption conditions for the removal of malachite green dye when LS/PANI composites were used as malachite green dye adsorbents were as follows: an adsorbent dosage of 20 mg, an initial concentration of the dye of 250 mg/L, an adsorption time of 300 min, and a temperature of 358 K. The LS/PANI composite adsorbed malachite green dye in accordance with the Langmuir adsorption model and pseudo-second-order kinetic model, which belongs to chemisorption-based monomolecular adsorption, and the equilibrium adsorption amount was 245.75 mg/g. In particular, the adsorption of heavy metal ion Pb^2+^ was investigated, and the removal performance was also favorable for Pb^2+^.

## 1. Introduction

The textile industry plays an important role in modern industrial production. China is the world’s largest exporter of textiles, and its export volume is higher than that of India, America, and other countries [1]. Overall, 80% of the total emissions from the textile industry affect water resources and increase the chemical oxygen demand, thereby inhibiting plant photosynthesis and impairing the welfare of aquatic organisms [2]. Dyes can be divided into natural dyes and synthetic dyes, and synthetic dyes are widely used because of their bright colors, wash resistance, and ability to be produced in large quantities [3]. Among them, azo dyes are the most widely used and the highest-yielding synthetic dyes [4]. The distribution of dyes in wastewater is positively correlated with the molecular weight of azo dyes. Said et al. [5] suggest that the molecular weight of azo dyes increases in the form of increasing azo bonds, which makes it difficult to purify water in the back-end process. The carcinogenicity and genotoxicity of these dyes will have a significant impact on the environment and human beings [6]. In addition, industrial wastewater contains a large number of heavy metal ions, which are not easily degradable, are toxic, and are extremely harmful to the environment and human beings (for example, Pb^2+^ is extremely harmful to humans, usually involving the immune system, affecting the nervous system, and so on) [7,8]. Therefore, there is an urgent need for the environmentally sound treatment of dyestuffs and industrial wastewater. For the removal of dyes from polluted water, there are various biological, chemical, and physical methods [9]. Biological methods include enzyme degradation, fungal culture, etc. Biodegradation methods, although very low-cost, have poor treatment efficacy [10]. Chemical methods, including ozonation, oxidation, electrochemistry, etc., have some problems, such as expensive reagents, high requirements for physical and chemical conditions, and secondary pollution [11]. Physical methods include adsorption, ultrafiltration, coagulation and flocculation, membrane separation, etc. The physical method is the most commonly used treatment method, with a low cost, high efficiency, and the smallest amount of chemicals used [10,12]. Among the physical methods, adsorption is the cheapest, most effective, and most popular method to remove organic dyes from polluted water [13,14]. Adsorption is classified into two types: chemisorption and physisorption [15]. Common adsorption materials are activated carbon, molecular sieves, ion exchange resin, and adsorption resin [15]. Among the many polymer adsorbents, polyaniline (PANI) has been widely used [16].

Polyaniline is a conductive polymer with good thermal stability and environmental stability, adjustable performance, easy doping and synthesis, a low cost, and a large surface area [16,17,18], as well as many application possibilities [19]. Its structure contains benzene and quinine rings, and its conductive state contains a considerable number of active groups, such as amine groups and imine groups, which provide PANI with good adsorption capacity [20], and it can treat different dye wastewaters. However, PANI particles are easily aggregated in a water medium, and the intermolecular and intramolecular interactions will reduce the surface area and limit the practical application [17]. PANI also has the problem of poor mechanical strength, which represents a great barrier in the industrialization of PANI as an adsorbent [21]. To overcome all these defects, researchers have investigated polyaniline composites by doping different materials into the PANI matrix, hoping to obtain materials with excellent physical and chemical properties.

Lignocellulose is the most abundant biomass resource. Lignin has impressive characteristics, such as high abundance, a high molecular weight, oxidation resistance, bacteriostasis, and biodegradability, and has great potential for adsorption (e.g., organic dyes, metal ions) [22]. A large number of hydroxyl groups create significant adsorption sites in lignin, and, at the same time, due to the carboxyl and phenol groups in its structure, lignin has a strong ability to remove heavy metal ions [23]. Composites of lignin or lignin derivatives with organic and inorganic materials have been reported to improve the adsorption capacity of composites, e.g., lignin/graphene composites, lignin/magnetic nanoparticle composites, lignin/carbon nanotube composites, and lignin/chitosan composites [22,24,25].

For this reason, we propose a lignosulfonate/polyaniline composite (LS/PANI) adsorbent. The structure of polyaniline can be effectively improved by introducing lignosulfonate molecules with three-dimensional networks, thus enhancing its adsorption properties. Lignosulfonate has good biocompatibility, reproducibility, and a high sulfonation degree [26]. Compared with PANI, LS/PANI adsorbents have enhanced conductivity [27] and mechanical flexibility [28], which substantially improves the adsorption performance of LS/PANI for organic dyes and heavy metal ions [29]. In this work, lignin-based porous composites were prepared from lignin, an industrial waste product from pulp and paper making, and were structurally and morphologically characterized using FTIR (Fourier Transform infrared spectroscopy), SEM (Scanning Electron Microscope), and other techniques. PANI and LS/PANI were used as adsorbents to investigate the effects of conditions such as adsorbent addition and the initial concentrations on the repellent performance of cationic dyes (malachite green (MG), methylene blue (MB), and crystal violet (CV)) and the heavy metal ion Pb^2+^. Models such as pseudo-first-order and pseudo-second-order kinetics were used to analyze the adsorption process of cationic dyes by kinetic fitting; the adsorption process of cationic dyes by thermodynamic fitting was also analyzed using isothermal adsorption models such as Langmuir and Freundlich, and the related kinetic and thermodynamic parameters were calculated.

## 2. Results and Discussion

### 2.1. Effect of Adsorbent Input, pH, and Initial Concentration on the Adsorption Performance of Dyes and Adsorption Capacity of LS/PANI for Different Dyes

**Figure 1:**
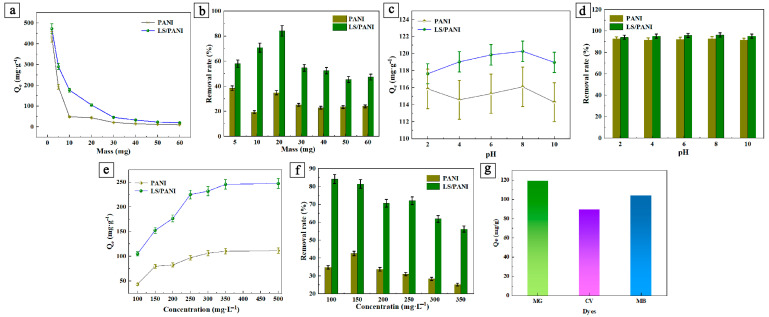
(**a**) Influence of adsorbent amount on adsorption capacity of MG; (**b**) effect of adsorbent amount on removal rate of MG; (**c**) influence of pH on adsorption capacity of MG; (**d**) influence of pH on removal rate of MG; (**e**) influence of initial concentration on adsorption capacity of MG; (**f**) influence of initial concentration on removal rate of MG; (**g**) adsorption capacity of LS/PANI for different cationic dyes.

The analysis of LS/PANI and PANI, as effective adsorbent materials in the adsorption of dyes such as malachite green, involves two indicators: the adsorption capacity and the removal rate. The adsorption capacity usually refers to the maximum amount of dye that can be adsorbed per unit mass of adsorbent under certain conditions. This indicator directly reflects the adsorption capacity of the adsorbent for the dye. The removal rate is the percentage decrease in the concentration of adsorbed dye in water during the adsorption process. Although both indicators describe the adsorption effect, they focus on different aspects. The adsorption amount focuses on the performance of the adsorbent material, while the removal rate is more reflective of the quality of the treated water. Therefore, tests of the adsorption capacity and removal rate are equally important. As can be seen from Figure 1a,b, for the malachite green dye, when the adsorbent dosage was less than 20 mg, the adsorption and removal rate increased rapidly with the increase in the adsorbent dosage. The removal rate of malachite green in PANI was about 40% at the dosage of 20 mg, and the removal rate in LS/PANI reached 84%. With the increase in adsorbent addition, the removal rate of malachite green decreased to a certain value and then remained almost unchanged. The reason for this phenomenon is that the increase in the adsorbent dosage provides more adsorption sites for the adsorption of the dye, which leads to an increase in the removal rate, and the adsorption amount of the dye per unit mass of adsorbent decreases because the initial concentration of the dye remains unchanged and decreases with the increase in the removal rate. In addition, higher adsorbent content also occurs with the agglomeration phenomenon in the solution, which is unfavorable for the adsorption process. To summarize, when the amount of LS/PANI adsorbent was 20 mg, the adsorption and removal of malachite green reached a maximum of 84.18%, which was much higher than the removal in PANI.

The pH of the solution is highly important in the adsorption of malachite green dye, as an overly high/low pH will produce changes in the functional groups of LS/PANI and MG, thus affecting the actual adsorption effect [30]. In order to examine the effect of pH on the removal of malachite green by PANI and LS/PANI composites, the initial pH range of 2–10 was used as a variable condition for the experiments. As can be seen in Figure 1c,d, 96.22% removal was achieved in the malachite green solution at pH = 8. This may be due to the fact that when in an acidic medium, both LS/PANI and MG are positively charged and both repel each other, which is not favorable for adsorption. Under alkaline conditions, the H^+^ in the solution decreases, the negative charge increases, and there is electrostatic attraction between the two. In particular, pH > 8.0 increases the repulsion between the two due to the protonation of certain functional groups [31]. The maximum adsorption capacity of the adsorbent was 120.27 mg/g in a partially alkaline solution with an initial concentration of malachite green dye of 100 mg/L at pH = 8. A decrease in the adsorption capacity was observed at either higher or lower pH levels, which may be attributed to the fact that the dye diffuses from the solution to the surface of the adsorbent during the first stage of adsorption. A low pH can severely damage the insolubility of MG within the water, while a high pH also affects the adhesion of the adsorbent to MG.

The initial dye concentration is an important factor to be considered in the effective adsorption process. In order to investigate the effect of different initial concentrations, a 100 mg/L stock solution was prepared by dissolving 0.1 g of MG dye in 1000 mL of distilled water, and different initial concentrations of the dye were prepared by dilution. Then, 20 mg of the sample was placed into the solution, and the other influencing factors remained unchanged (pH = 6, 25 °C). The study of different initial dye concentrations can help to understand the behavior of the adsorption process and optimize the efficiency of the adsorbent’s use and the design of the adsorption process, in order to provide valuable information for the treatment of dye wastewater of different concentrations. The influence relationship between the adsorption amount and removal rate of the dye solution and the initial concentration is shown in Figure 1e,f. From the figure, it can be seen that the adsorption amount of malachite green is highly dependent on the initial concentration of the dye; with the increase in the initial concentration of the dye, the adsorption amount of the adsorbent shows an increasing trend and finally levels off (higher concentrations of 500 mg/L). When the initial concentration of malachite green is 250 mg/L, the increase in the adsorption amount of the dye is almost insignificant, while the removal rate of the dye decreases. This result can be attributed to the fact that the initial concentration of the dye provides an important driving force to overcome the mass transfer resistance of the dye between the aqueous and solid phases [32]. Therefore, a higher initial dye concentration enhances the adsorption process, and the higher the initial concentration of dye, the better the adsorption effect, until the saturated adsorption concentration is reached and the amount of adsorption tends to equilibrate. From Figure 1e, it can be seen that the maximum adsorption of malachite green was 245.75 mg/g and it then leveled off, while the maximum adsorption of malachite green by PANI could only reach 85.02 mg/g, which was much less effective than the LS/PANI composites.

The MG adsorption capacity was 119.4 mg/g, while the CV adsorption capacity was 89.5 mg/g and the MB adsorption capacity was 104.3 mg/g, in a solution at an initial concentration of 100 mg/L and pH = 7 (Figure 1g). It can be found that the adsorption capacity of LS/PANI on MG, MB, and CV is not much different, probably because they have similar structures and molecular weights.

### 2.2. Adsorption Isotherms and Thermodynamic Analysis

When the adsorbent material reaches the adsorption equilibrium, there is a relationship between the equilibrium adsorption amount at this time and the equilibrium concentration of the dye adsorbed, and the theoretical calculation of the adsorption thermodynamic data can be used to deduce the maximum adsorption amount of the adsorbent, as well as the possible adsorption mechanism. The Langmuir and Freundlich isotherm models are often used to study the relationship between adsorbent uptake and the adsorbent material’s surface. In order to investigate the adsorption mechanism of LS/PANI composites in depth, the thermodynamic parameters of the adsorbed dyes were investigated in this study using these two models (Freundlich isotherm model and Langmuir isotherm model) [33]. The Langmuir model is applicable to the case of monolayer adsorption, while the Freundlich model is applicable to the case of multilayer adsorption.

Freundlich concluded that adsorption can occur by the formation of a multilayer film on the inhomogeneous surface of the adsorbent. The Freundlich isothermal adsorption model equation is as follows (Equation (1)):

(1) $$\ln Q_{e}=\ln K_{F}+\frac{1}{n}\ln C_{e}$$

where Q_e_—equilibrium adsorption amount, mg/g;
C_e_—equilibrium concentration, mg/L;
K_F_—adsorption equilibrium constant;
n—intensity factor.

Langmuir considered the formation of a monomolecular layer on the surface of the adsorbent; the other refers to the adsorption of adsorbent with only one molecule at an adsorption site, resulting in a decrease in the intermolecular force with increasing distance [34]. The aforementioned isotherms assume that the adsorbent surface is uniform, with similar as well as potentially equivalent adsorption sites [35]. The Langmuir isothermal adsorption model equation is as follows (Equation (2)):

(2) $$\frac{C_{e}}{Q_{e}}=\frac{1}{K_{L}Q_{m}}+\frac{C_{e}}{Q_{m}}$$

where K_L_—Langmuir’s constant, L/mg;
Q_m_—the maximum adsorption capacity per unit mass of adsorbent, mg/g.

**Figure 2:**
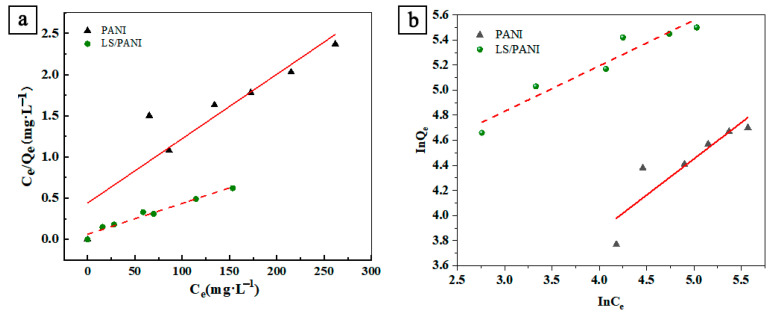
(**a**) Langmuir model of MG linear fitting; (**b**) Freundlich model of MG linear fitting.

The correlation parameters of the Langmuir and Freundlich adsorption models for malachite green dye are shown in Table 1, and the linear fits of the Langmuir model and Freundlich model for malachite green dye are shown in Figure 2a,b, respectively. The correlation coefficient of the Langmuir model for malachite green dye adsorption was higher than that of the Freundlich adsorption isothermal model, the fitting accuracy was higher in the Langmuir isothermal model, and the process involved the adsorption of a single molecular layer. The comparison of Figure 2a,b shows that the linear fits yield similar fitting results. The theoretical maximum adsorption capacity of the Langmuir model is greater than the actual adsorption capacity, and the reason for the large theoretical adsorption capacity is that the adsorption capacity may increase further as the initial concentration increases until the adsorption equilibrium is reached.

### 2.3. Effect of Adsorption Temperature on Dye Adsorption Properties and Thermodynamic Analysis

**Figure 3:**
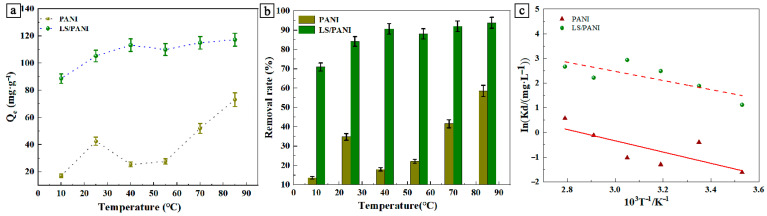
(**a**) Influence of temperature on the adsorption capacity of MG; (**b**) effect of temperature on removal rate of MG; (**c**) adsorption of malachite green on InK_d_ and 1/T curves.

The effects of different temperatures on the adsorption of dyes were investigated and the optimal adsorption temperature conditions were determined. Temperature gradients of 85 °C, 70 °C, 55 °C, 40 °C, 25 °C, and 10 °C (i.e., 358, 343, 328, 313, 298, and 283 K) were set, and the adsorption equilibrium was reached by adding 20 mg of the sample to a malachite green solution with pH = 6 and 25 mL of an initial concentration of 200 mg/L, respectively. Figure 3a shows the adsorption of malachite green in the PANI and LS/PANI adsorbent materials at different temperatures, and Figure 3b shows the removal rate effect. From Figure 3a, it can be seen that the adsorption amount of malachite green in the LS/PANI adsorbent materials increased with the increase in the adsorption temperature, and the trend of change was the same, indicating that the adsorption process of the dye was consistent with the mechanism of the temperature change. The effect of the LS/PANI material in the removal of the dye was significantly better than that of PANI, especially when the PANI was used to treat the malachite green dye, wherein the amount of adsorption was small. In order to further explore the mechanism of this process, thermodynamically relevant parameters were used.

(3) $$K_{d}=\frac{{\mathrm{mq}}_{e}}{C_{e}V}$$

(4) $$\ln K_{d}=\frac{{\Delta S}^{0}}{R}-\frac{{\Delta H}^{0}}{\mathrm{RT}}$$

(5) $$\Delta G^{0}=\Delta H-T\Delta S$$

where R—standard molar constant, 8.314 × 10^−3^ J/(mol·K);
ΔG^0^—Gibbs free energy, kJ/mol;
ΔS^0^—standard entropy change, kJ/(mol·K);
ΔH^0^—standard enthalpy change, kJ/mol;
K_d_—partition coefficient;
m—mass of adsorbent, g;
V—volume of dye solution, L.

Table 2 shows the PANI and LS/PANI adsorbed MG thermodynamic parameters. From the results in Table 2, ΔG^0^ < 0 in the adsorption of MG dye by LS/PANI indicates that it is a positive spontaneous process, while, in PANI, contrary to the other adsorption processes, the extent of the reaction is smaller, and the rate of adsorption is so low that the reaction does not proceed spontaneously. ΔH^0^ > 0 proves that the adsorption is a heat-absorbing reaction, and the adsorption of the dye by the adsorbent is an irreversible heat-absorbing process [36]. Meanwhile, ΔS^0^ > 0 indicates that the adsorption process is an entropy-increasing process, which may be due to the increase in the number of molecules or ions after adsorption, resulting in an increase in disorder at the interface of the solid–liquid phases [37]. In addition, the LS/PANI material decreased from −3.50 kJ/mol to −8.51 kJ/mol in malachite green as the adsorption temperature was increased from 283 K to 358 K. It can be seen that as the temperature increases, the system of matter will reach the equilibrium state more easily, so the free energy decreases. This indicates that at higher temperatures, it is favorable for the adsorption process to obtain higher adsorption equilibrium capacity spontaneously, which enables the dye molecules to move rapidly, thus maximizing the adsorbable number of active sites and subsequently enhancing its adsorption capacity [38]. At a higher adsorption temperature, in combination with the Langmuir isothermal model, the increase in temperature allows more dye molecules to be activated, thus increasing the chemical equilibrium constant in the adsorption process. Thus, the maximum adsorption of malachite green of 117 mg/g can be obtained at the temperature of 358 K. As the temperature increases, the kinetic energy of the dye molecules increases, which will help the dye to diffuse.

### 2.4. Effect of Adsorption Time on Dye Adsorption Properties and Adsorption Kinetics

**Figure 4:**
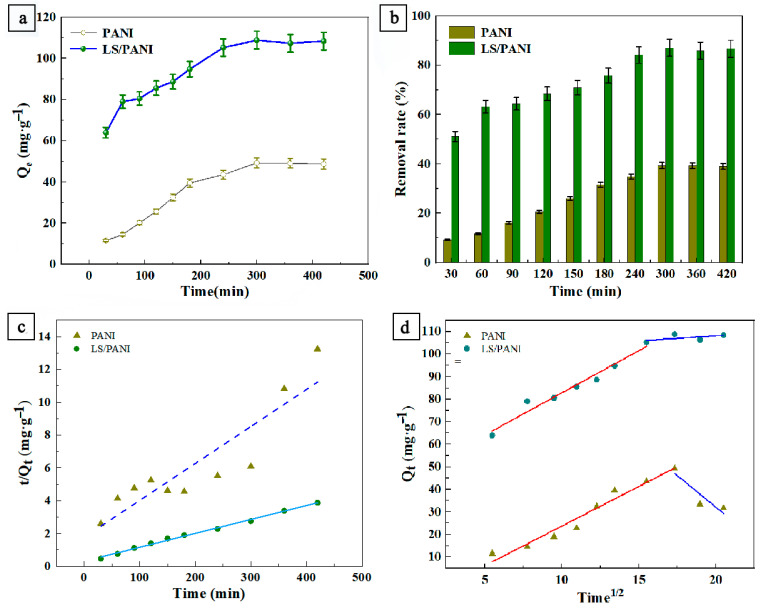
(**a**) Influence of time on the adsorption capacity of MG; (**b**) effect of time on removal rate of MG; (**c**) pseudo-second-order kinetic model of MG; (**d**) intra-particle diffusion model of MG (the red and blue lines represent the different adsorption stages fitted).

The effect of the adsorption time on the amount of dye adsorbed is shown in Figure 4a,b. From Figure 4a, it can be seen that LS/PANI adsorbed malachite green at 51.12% at the beginning of adsorption for 30 min. At this stage, the larger specific surface area of LS/PANI produces higher surface energy, so that there is a large number of vacant active sites on the surface, coupled with the rapid migration of dye molecules to these sites in the solution. At 300 min, for malachite green dye, the rate of adsorption of MG began to level off, the adsorption reached an equilibrium, and the diffusion of malachite green dye from the surface of the adsorbent to the internal pores was more time-consuming. At 300 min, it reached the adsorption equilibrium state. At this time, pore diffusion dominated, and the maximum adsorption of LS/PANI in malachite green was up to 108.34 mg/g, which was much higher than that of PANI without added lignin (49.28 mg/g).

Kinetic studies help to elucidate the optimal time at which adsorption equilibrium is reached, which is important in analyzing the adsorption mechanism during adsorption. The efficiency of the adsorbent depends not only on the adsorption capacity but also on the rate of uptake of pollutants from the wastewater [33]. Generally, the adsorption procedure can be classified into three primary phases. In the first phase, MG spreads from the liquid to the adsorbent interface (membrane spreading phase). In the second phase, MG enters the internal pores from the PANI and LS/PANI surfaces (intra-particle diffusion phase). The third phase involves the adsorption of the sorbent on the adsorption sites. During these three phases, the mass transport resistance of the adsorbent is different, and the overall rate of the adsorption process is determined by the largest mass transport resistance and the smallest step size. The adsorption equilibrium will be rapidly established on the adsorption site [39]. In this study, the adsorption kinetics of malachite green were studied from 0 to 420 min (other influencing factors were kept constant), and the adsorption kinetics and equilibrium parameters were determined to understand the adsorption mechanism of PANI and LS/PANI.

The kinetics of MG were further investigated based on the data from the pseudo-first-order and pseudo-second-order kinetic models, which are shown in Equations (6) and (7), respectively. Based on the pseudo-first-order kinetic model (Freundlich isothermal adsorption model) and the pseudo-second-order reaction-controlled kinetic model (Langmuir isothermal adsorption model), respectively, the adsorption processes of the two materials, PANI and LS/PANI, were analyzed.

Pseudo-first-order kinetic model:

(6) $$\ln(q_{e}-q_{t})=\ln q_{e}-k_{1}t$$

where q_e_—equilibrium adsorption amount, mg/g;
k_1_—adsorption rate constant, min^−1^;
q_t_—adsorption amount at time t, mg/g.

Pseudo-second-order kinetic model:

(7) $$\frac{t}{q_{t}}=\frac{1}{k_{2}q_{e}^{2}}+\frac{t}{q_{e}}$$

where q_e_—equilibrium adsorption amount, mg/g;
k_2_—adsorption rate constant in this model, g/mg/min;
q_t_—adsorbed amount per unit mass of adsorbent at any adsorption time t, mg/g.

Particle diffusion equation:

(8) $$q_{t}=k_{d}t^{\frac{1}{2}}+C$$

Figure 4c,d show the linear fitting plots of the pseudo-second-order kinetic model and intra-granular diffusion model for MG. Table 3 shows the parameters related to the pseudo-first-order and pseudo-second-order kinetic models for the adsorption of malachite green. From Figure 4a, it can be seen that the adsorption of MG increased rapidly with the increase in the adsorption time; subsequently, the rate of increase in adsorption gradually slowed down until equilibrium was reached. The pseudo-first-order kinetic equation assumes that the adsorption process is controlled by diffusion, and the pseudo-second-order kinetic equation assumes that the adsorption rate is controlled by the chemisorption mechanism. It can also be seen from Table 3 that after kinetic model fitting, the pseudo-second-order kinetic model fitted by MG showed a better linear relationship, the pseudo-second-order adsorption kinetic-fitted values of both the PANI and LS/PANI materials in malachite green were larger, and the adsorption process was dominated by chemisorption. After fitting, the equilibrium adsorption amounts of 83.95 mg/g and 113.70 mg/g of malachite green by PANI and LS/PANI were more consistent with the actual adsorption amounts of 49.28 mg/g and 108.79 mg/g. The high simulated correlation coefficients indicated that the adsorption process of malachite green by PANI and LS/PANI was similar to the pseudo-second-order kinetic model. Chemisorption plays a major role in the adsorption process, which involves electron transfer or electron pairing between the adsorbent molecules and the adsorbed agent. In summary, LS/PANI reached the saturation adsorption of malachite green at 108.79 mg/g when the adsorption time was 300 min.

Table 4 shows the parameters related to the internal diffusion model for the adsorption of malachite green. As can be seen from Table 4, K_i1_ > K_i2_ indicates that fast adsorption occurs in the initial stage, which is mainly controlled by boundary layer diffusion, while the slow adsorption in the later stage is mainly controlled by intra-particle diffusion. Meanwhile, it was found that C_1_ < C_2_. Not only was the adsorption rate higher in the first stage, but also the adsorption amount had a significant advantage, and the sum values of LS/PANI were all larger than those of PANI. The higher R^2^ in the first stage indicated that the proposed model had better predictability, and the fitted straight lines of both PANI and LS/PANI did not pass through the origin, which indicated that the straight-line rate control process of adsorption to MG was not controlled only by intra-particle diffusion [33,36].

Due to the similarity of the chemical structures of malachite green and LS/PANI (both contain conjugated molecular structures and aromatic rings), the benzene ring contained in LS/PANI may form a π–π interaction with the benzene ring on the dye molecule to adsorb the organic pollutants. The addition of LS adds more sulfonic acid groups and hydroxyl groups, and the hydroxyl groups have an electron-withdrawing effect, which increases the adsorption sites, causing an interaction with the dye. The adsorption amount was obviously increased by the addition of LS, which increased the sulfonic acid groups and hydroxyl groups. The amine and imine groups in the composites may form intermolecular hydrogen bonds with the dye molecules. Analyzing the structure of MG, polyaniline has a positive charge on the main chain. In sp^3^ hybridization, a -NH_2_ lone pair of electrons is present, which can form a coordination bond with malachite green cation molecules to trigger adsorption, but its adsorption effect is significantly weaker than the electrostatic adsorption effect. In addition, the cations ionized in water will also produce a certain electrostatic repulsion effect with positively charged ions, which is not conducive to the increase in adsorption.

### 2.5. Mechanism of Adsorption Performance and Recycling Performance

The SEM and TEM (Transmission electron microscope) images of LS/PANI and PANI are shown in Figure S1a–f. Pure polyaniline is a distinct fibrous strip with irregular structural crossings. In the LS/PANI composite, the introduction of LS caused a great change, and the original fiber structure was “coarsened” into a nanosphere structure with well-developed pores. The addition of LS provides multiple active sites and pore channels for the interaction between aniline monomers, which will facilitate the adsorption of molecules and ions in LS/PANI composites. Therefore, it is said that the doping of LS enhances the adsorption capacity of PANI. Figure S1g shows the N_2_ adsorption and desorption curves of LS/PANI. The specific surface area of LS/PANI is 32 m^2^/g. The average pore size of PANI is 3.23 nm, and the average pore size of LS/PANI is 23.88 nm. The composites have a three-dimensional mesh fibrous globular structure, which is characterised by its porosity, and its specific surface area is larger. The addition of LS causes the molecular chains in PANI to form a uniform coating structure, which is conducive to the transfer of dye molecules and heavy metal ions in and between the chains, thus improving the adsorption capacity. Figure S1h is the full XPS spectrum of LS/PANI with PANI, showing the successful doping of S as well as the N-element of polyaniline itself. Figure S1i,j show the nitrogen-containing surface functional groups of the composites along with the sulfur-containing surface functional groups, and the negatively charged sulphonic acid groups in LS also act as anions/dopants. In the equilibrium oxidation state, the PANI molecule is positively charged and the hydrogen on its groups can dissociate and form intramolecular ionic bonds, leading to the formation of a conductive semiquinone-doped state of PANI, which would be favorable for the adsorption of the composite.

**Figure 5:**
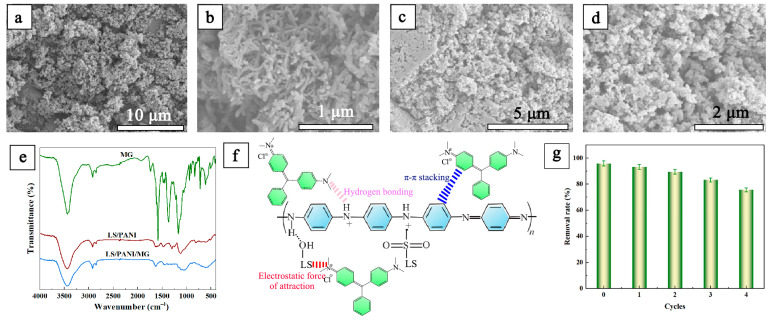
(**a**,**b**) SEM images of LS/PANI; (**c**,**d**) SEM image of LS/PANI after adsorption of MG; (**e**) FTIR spectra of MG, LS/PANI, and LS/PANI/MG; (**f**) mechanism diagram of assumed LS/PANI adsorption of MG; (**g**) recycling effect diagram of LS/PANI adsorption MG.

To further analyze the adsorption mechanism of LS/PANI on MG and to select the optimal adsorption conditions for the adsorption of the dyes, the micro-morphological changes of the adsorbent before and after adsorption were characterized by SEM. The LS/PANI before adsorption consisted of aggregates of nanorod-like fibers of different lengths (Figure 5a,b) [40], and the morphology of the complexes changed considerably after the adsorption of malachite green, as shown in Figure 5c,d. After the adsorption of malachite green, the dye molecules occupied a large number of active sites, a layered spherical morphology of about 250 nm was formed, and the nanocomposites’ surface porosity increased. The significant change in the original fibrous surface morphology proves the successful loading of malachite green dye on the surfaces of the nanocomposites.

The surface groups of the adsorbent were characterized using FT-IR, and the IR spectra of MG and LS/PANI before and after adsorption were obtained, as shown in Figure 5e, which confirms the adsorption of MG on LS/PANI. Several typical absorption characteristic peaks can be observed after the adsorption of LS/PANI/MG. In addition, the absorption peaks at 1461 cm^−1^ and 1631 cm^−1^ correspond to the backbone vibration of the C=C quinone ring and benzene ring. This spectral band underwent obvious displacement and intensity enhancement and showed a sharper peak shape after adsorption, which may be attributed to the enhancement of backbone vibration in the LS/PANI adsorbent due to the influence of the structure of MG during the adsorption process. The characteristic peak at 1313 cm^−1^ reflects the backbone vibration with the benzene ring connected to the stretching vibration of secondary amine C-N. Compared with LS/PANI, the spectral peaks of LS/PANI/MG after adsorption show a significant blue shift at 806–500 cm^−1^. The absorption peaks around 1120 cm^−1^ in the main chain of LS/PANI/MG are the planar bending vibration of C-H in the protonation process of the N=Q=N structure of the quinone ring. The absorption peak around 1120 cm^−1^ in the main chain of LS/PANI/MG is the C-H planar bending vibration of the quinone ring in the structure of N=Q=N during the protonation process, and the absorption peak around 806 cm^−1^ is the absorption peak of the 1,4-substituted benzene ring in LS/PANI. The spectral peak is broadened by the adsorption of malachite green [28,29,41]. The FT-IR spectra of the composites changed to some extent after the adsorption of malachite green, indicating that the adsorbent showed chemical adsorption in addition to the removal of malachite green by physical adsorption, which was consistent with the model predictions. Therefore, the main adsorption mechanisms of LS/PANI on MG are electrostatic interaction, hydrogen bonding, and π–π interaction (Figure 5f). One of the most important factors in the adsorption process is the regeneration of the adsorbent in order to increase its economic efficiency. Figure 5g shows a comparison of the adsorption effect of LS/PANI under desorption and regeneration cycles. Regeneration experiments with LS/PANI were performed, in which LS/PANI was regenerated with 0.1 M HCl and 0.1 M NaOH solution as a combined eluent [31]. The removal of MG decreased due to the decrease in the active sites of LS/PANI as the cycle proceeded. After four regenerations, the removal rate of MG was 75.7%, which indicates that LS/PANI has a good regeneration ability.

### 2.6. Adsorption of Heavy Metal Ions by LS/PANI and Comprehensive Performance Evaluation

**Figure 6:**
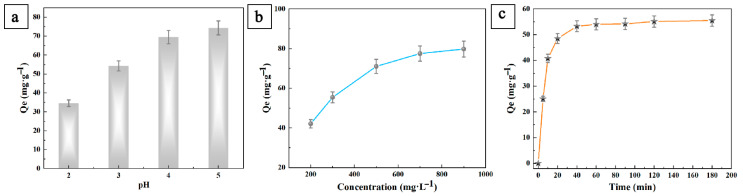
(**a**) Influence of pH on removal rate of Pb^2+^; (**b**) influence of initial concentration on adsorption capacity of Pb^2+^; (**c**) influence of adsorption time on removal rate of Pb^2+^.

We also applied LS/PANI to heavy metal adsorption. The effects of the initial Pb^2+^ concentration, pH value, and adsorption time on the adsorption performance of Pb^2+^ were investigated. The Pb^2+^ adsorption capacity showed an increasing trend with the increase in the initial concentration of Pb^2+^, and the increase in the Pb^2+^ adsorption capacity varied less when the initial concentration of Pb^2+^ was around 700 mg/L (Figure 6b). The Pb^2+^ adsorption capacity of LS/PANI reached 74.37 mg/g at pH = 5 (Figure 6a). When the pH value is relatively low, the amino group and imine group in LS/PANI are protonated, the polymer chain is positively charged, and the polymer chain is electrostatically repulsed by the Pb^2+^. When the pH value is increased, the amino group and imine group in LS/PANI are deprotonated to a larger extent, and the complex adsorption ability of the amino group and imine group of the molecular chain of LS/PANI with the heavy metal ions is increased, so the adsorption capacity of Pb^2+^ increases continuously. The adsorption capacity of Pb^2+^ gradually decreases at pH > 5 and Pb(OH)_2_ precipitation is formed, after which the adsorption capacity further decreases. When the solution is neutral or alkaline, a Pb(OH)_2_ precipitate is gradually formed and the adsorption capacity decreases [42,43]. As shown in Figure 6c, the adsorption of Pb^2+^ by LS/PANI varied with time. In the first 40 min of adsorption, the adsorption curves all showed a rapid growth trend, and the adsorption amount of Pb^2+^ by LS/PANI changed little after 40 min. When the adsorption reached 40 min, the removal rate of Pb^2+^ reached 55.48 mg/g.

With the aim of comparing the adsorption performance of LS/PANI composites, Table 5 shows the saturation adsorption of different adsorbents and LS/PANI composites. As the results show, the saturation adsorption of malachite green and the adsorption of heavy metal ions by LS/PANI composites were equal or even higher than the saturation adsorption of other lignin composites. The above results indicate that the LS/PANI composites prepared in this work have good adsorption performance for organic pollutants and heavy metal ions.

## 3. Materials and Methods

### 3.1. Materials

Lignosulfonate, methylene blue, malachite green, crystal violet, and Pb^2+^ standard solution were obtained from Aladdin Reagent Co. (Shanghai, China). Aniline and ammonium persulfate were purchased from Macklin Reagent Co. (Shanghai, China). HCl was obtained from Nanjing Chemical Reagent Co. (Nanjing, China). Ultra-pure water was obtained from a laboratory device (Nanjing, China).

### 3.2. Synthesis of Adsorbent Materials

**Figure 7:**
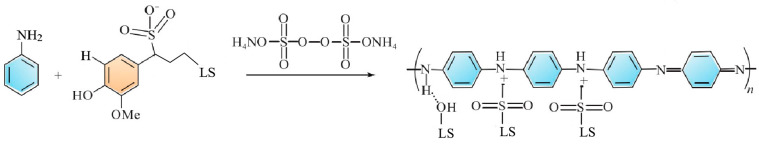
Mechanistic diagram for the synthesis of lignosulfonate/polyaniline.

LS/PANI composites were obtained from the previous work of our group, prepared according to the literature using chemical oxidative polymerization (Figure 7) [41]. First, 0.10 g of LS was dissolved in 50 mL of 1.0 M HCl and subjected to ultrasonic treatment for a period of time. To the above-mentioned solution, 0.91 mL of aniline was fed and it was stirred at a low temperature of 0 °C for 2 h. Then, 2.30 g of ammonium persulfate solution (APS) was dissolved in 50 mL of 1.0 M HCl. A 1:1 (mol:mol) mixture of aniline and APS was then subjected to a polymerization reaction at 0 °C for 24 h. At the end of the reaction, the reaction product was filtered, and the slag was washed several times with diluted water until neutral. It was then freeze-dried for 48 h and then dried under a vacuum to a constant quality to obtain the LS/PANI composite. The PANI was reacted without the addition of LS in the reaction step.

### 3.3. Characterization and Adsorption Conditions

A field emission scanning electron microscope (Quanta 20, Tokyo, Japan) was used to observe the microscopic morphological characteristics of LS/PANI and PANI. Before the SEM test, the black powder sample was attached to the conductive adhesive of the sample table and sprayed with gold treatment to eliminate static electricity. The samples were analyzed on a VERTEX 80 V infrared spectrometer in the wavelength range of 4000–400 cm^−1^ using the KBr compact method.

The characterization of the physical structure and morphology of PANI and LS/PANI is shown in the Supporting Materials, Figure S1. Additional characterization conditions and the detailed steps of the adsorption experiments can also be found in the Supporting Materials. The molecular structure of malachite green can be found in Figure S2 in the Supporting Materials. The concentration of malachite green was measured by an ultraviolet spectrophotometer (UV2200, Shanghai Sunyu Hengping Scientific Instrument Co., Ltd., Shanghai, China), and the standard curves for different concentrations of malachite green can be found in the Supporting Materials, Figure S3.

To investigate the adsorption performance of the two adsorbents, PANI and LS/PANI, a single variable method was adopted for different pH values and adsorbent dosages.

For the adsorption performance under different pH conditions, 0.1 mol/L HCl and 0.1 mol/L NaOH were used to adjust the pH in the experiment. First, 20 mg of adsorbent, malachite green solution with a concentration of 100 mg/L, and pH values of 2, 4, 6, 8, 10, and 12 were used in a conical flask, and the adsorption temperature was 25 °C. The adsorption was carried out with 0.1 mol/L HCl and 0.1 mol/L NaOH [33]. After adsorption, the supernatant was filtered by a 0.22 μm filter membrane, and the removed supernatant was tested for absorbance by a UV spectrophotometer; if the absorbance was too high, it was diluted and then its absorbance was measured again.

For the effect of the adsorbent dosage, the PANI and LS/PANI composites (10 mg, 20 mg, 30 mg, 40 mg, 50 mg, 60 mg) was dried to a constant weight and accurately weighed and placed in 25 mL of 100 mg/L malachite green dye solution. Then, they were placed in a thermostatic oscillator with a vibration speed of 150 rpm at 25 °C for 3 and 4 h. Then, the absorbance after adsorption was measured and the corresponding adsorption concentration was calculated from the standard curve, and the adsorbed amount and removal rate of the dye were calculated according to Equations (S1) and (S2).

Adsorption of heavy metals: In the first stage, the samples were placed in Pb^2+^ standard solution (which can be diluted to different concentrations according to the actual situation) for adsorption. Then, the Pb^2+^ concentration in the supernatant was detected using an inductively coupled plasma mass spectrometer (ICP-MS, iCAP RQ, Berlin, Germany). To ensure accuracy, each experiment was conducted in triplet. The formula for the adsorption capacity Q_e_ (mg/g) of LS/PANI can be calculated with reference to that of the dye above.

## 4. Conclusions

The LS/PANI composite material, which was produced from lignin, a paper waste product, was used as an effective dye adsorbent for the adsorption of cationic dyes and heavy metal ions. The adsorption of the malachite green dye by LS/PANI composites conformed to the Langmuir adsorption model and the pseudo-second-order adsorption kinetic model, and it showed chemisorption-dominated single-molecule adsorption, where the maximum adsorption amount was 250.01 mg/g. The adsorption mechanism also consisted of pore space adsorption and chemosorption. The whole adsorption process was entropy-increasing and spontaneous. In particular, the Pb^2+^ adsorption capacity of LS/PANI could also reach 74.37 mg/g at pH = 5. It is believed that the design of lignin materials with different coatings has great future potential, not only in the field of adsorbent materials but also in supercapacitors, novel solar energy batteries, and rechargeable batteries.

## Figures and Tables

**Table 1:** Parameters of Langmuir model and Freundlich model of MG.

Samples	Langmuir Isotherm Model			Freundlich Isotherm Model		
	$K_{L}$ $\;(g\cdot L^{-1})$	$Q_{m}$ (mg·g^−1^)	R^2^	$K_{F}$ $\;(mg^{1-1/n}\cdot L^{-1/n})$	n	R^2^
PANI	3.33 × 10^−3^	210.20	0.94	3.43	1.67	0.91
LS/PANI	2.76 × 10^−3^	513.36	0.98	6.11	1.57	0.96

**Table 2:** Thermodynamic parameters of adsorption of MG by PANI and LS/PANI.

Samples	$\Delta G^{0}/(kJ\cdot {mol}^{-1})$						$\Delta H^{0}(kJ\cdot {mol}^{-1})$	$\Delta S^{0}(J\cdot {mol}^{-1}\cdot K^{-1})$
	283 K	298 K	313 K	328 K	343 K	358 K		
PANI	3.66	2.85	2.03	1.22	0.41	−0.41	18.99	54.17
LS/PANI	−3.50	−4.50	−5.51	−6.51	−7.51	−8.51	15.43	66.89

**Table 3:** The parameters of pseudo-first-order kinetic model and pseudo-second-order kinetic model of MG.

Samples	Pseudo-First-Order Kinetic Model			Pseudo-Second-Order Kinetic Model		
	$Q_{1}$ $\;(\mathrm{mg}\cdot g^{-1})$	$k_{1}$ (min^−1^)	R^2^	$Q_{2}$ (mg·g^−1^)	$k_{2}$ (g·mg^−1^·min^−1^)	R^2^
PANI	58.02	5.31 × 10^−3^	0.95	83.95	4.80 × 10^−5^	0.96
LS/PANI	101.55	2.35 × 10^−2^	0.71	113.70	3.02 × 10^−4^	0.90

**Table 4:** Internal diffusion model parameters of adsorption of MG.

Samples	$K_{i1}$ (mg·g^−1^·min^−0.5^)	$C_{1}$ (mg·g^−1^)	$R_{1}^{2}$	$K_{i2}$ (mg·g^−1^·min^−0.5^)	$C_{2}$ (mg·g^−1^)	$R_{1}^{2}$
PANI	3.51	−11.40	0.96	−5.60	144.11	0.83
LS/PANI	3.74	45.42	0.96	0.42	99.51	0.30

**Table 5:** Comparison of adsorption properties of LS/PANI composites with other materials.

Lignin Composite Materials	Dye/Heavy Metal Ion Type	Adsorption Capacity (mg·g^−1^)	Ref.
Graphene oxide/aminated lignin aerogels	MG	113.50	[24]
Activated lignin/chitosan	MB	36.25	[25]
Chitosan/nano-lignin	MB	74.07	[44]
Chitosan/alkali lignin	Remazol Brilliant Blue R	111.11	[45]
Lignin–magnetic nanoparticle composite	Turquoise blue QG-125	72.83	[46]
Bio-based sodium alginate/lignin	MB	254.30	[47]
Porous graphene oxide/alkali lignin	MB	98.30	[48]
Enzymatic hydrolysis lignin/PANI	Ag^+^	662	[49]
Porous graphene/lignin/sodium alginate	Cd^2+^	79.88	[50]
	Pb^2+^	226.24	
Sodium lignosulfonate/lysine	Cu^2+^	365.10	[51]
TiO_2_/lignin	Pb^2+^	35.70	[52]
TiO_2_-SiO_2_/lignin	Pb^2+^	59.93	[52]
Lignin/MgO-SiO_2_	Cu^2+^	83.98	[53]
LS/PANI	MG/Pb^2+^	250.01/79.81	This work

## Data Availability

Data are contained within the article.

## Supplementary Materials

The following supporting information can be downloaded at: https://www.mdpi.com/article/10.3390/ijms25010133/s1.
