# Supplementary material for: Comprehensive Evaluation of Polyaniline-Doped Lignosulfonate in Adsorbing Dye and Heavy Metal Ions
Source: Int J Mol Sci. 2023 Dec 21;25(1):133. doi: 10.3390/ijms25010133 (PMC10779345; doi:10.3390/ijms25010133)
Supplement: Supplementary file 1 [file ijms-25-00133-s001.zip › ijms-2748502-supplementary.pdf]

Supporting Information

Comprehensive Evaluation of Polyaniline-Doped Lignosulfonate in Adsorbing Dye and Heavy Metal Ions

Wenjuan Wu [1,2,*], Penghui Li [1,2], Mingkang Wang [2], Huijun Liu [3], Xiufu Zhao [2], Caiwen Wu [1,2] and Jianpeng Ren [1,2]

[1] Jiangsu Co-Innovation Center of Efficient Processing and Utilization of Forest Resources, Nanjing Forestry University, Nanjing, 210037, P.R.China.

[2] College of Light Industry and Food Engineering, Nanjing Forestry University, Nanjing, 210037, P.R.China.

[3] School of Chemistry and Chemical Engineering, Southeast University, Nanjing 211189, P.R.China.

1 Material Characterization & Results

An AXIS Ultra DLD X-ray photoelectron spectrometer (XPS, Shimadzu, Japan) was used to determine the surface chemical composition of the samples, including details of the surface elemental composition and peaks of C (C 1s), O (O 1s), N (N 1s), and S (S 2p). The sample detection concentration is greater than 0.1%, the detection depth is less than 10 nm, at least two samples are prepared for each raw material, and at least three different positions are measured for each sample. Include the relative content of elements and the type and relative content of chemical functional groups. The N_2 adsorption and desorption isotherm data of samples were acquired using a Quantachrome Nova 2000e Surface and aperture analyzer (America) at low temperature and relative pressure P/P_0 = 0.001–1. The Brunauer–Emmett–Teller (BET) method and Barrett–Joyner – Halenda (BJH) theory were utilized to calculate the specific surface area and pore size distributions of samples, respectively. The surface morphology of the samples was characterized using transmission electron microscopy (TEM, JEM-1400, JEOL, Tokyo, Japan). The samples were suspended in ethanol prior to TEM testing, dripped onto a copper mesh microgrid, dried and used for observation, and the excitation voltage of the electron beam was 200 kV.

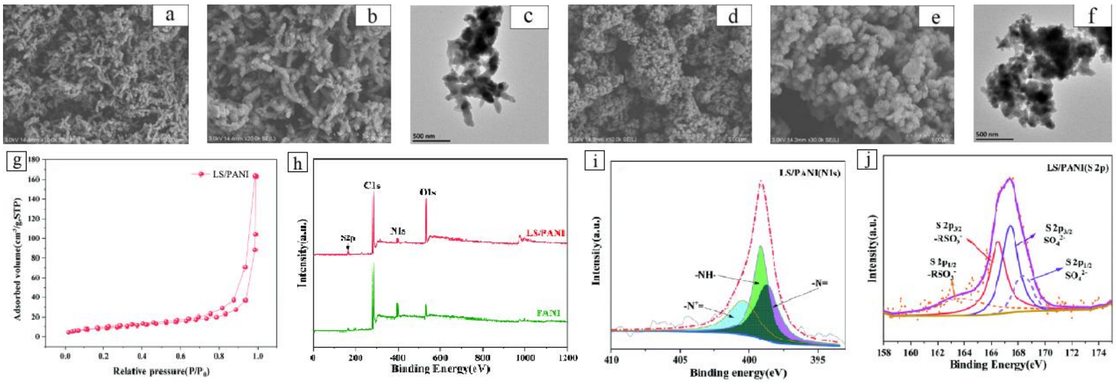

Figure. S1 SEM images of PANI (a) and (b) (magnification 10 000, 20 000 times), SEM images of LS/PANI (d) and (e) (magnification 10 000, 30 000 times), TEM images of PANI and LS/PANI (c) and (f); N_2 adsorption–desorption curves of LS/PANI (g); Full Spectrum of XPS of PANI and LS/PANI (h), XPS high-resolution spectra of N 1s (i) of LS/PANI, XPS high-resolution spectra of S 2p (j) of LS/PANI [30].

2 Adsorption measurement

2.1 Determination of maximum absorption wavelength

Malachite green (Molecular formula: $C_{23}H_{25}N_2Cl$, molecular weight: 364.5) is accurately weighed for the corresponding organic pollutant, and a 10 mg/L dye solution is accurately configured in volumetric flasks and prepared in different concentration gradients. Absorption or reflectance in the visible range directly affects the perceived color of the chemicals involved. The full spectrum of the organic pollutant solution can be scanned by an ultraviolet spectrophotometer (UV2200, Shanghai Sunyu Hengping Scientific Instrument Co., Ltd.) to determine the maximum absorption wavelength of the dye.

The maximum absorption wavelength of malachite green is 618 nm, the maximum absorption wavelength of methylene blue is 664 nm, and the maximum absorption wavelength of crystal violet is 590 nm, as determined by UV spectroscopy.

2.2 Determination of standard curve

Different concentration gradients of malachite green (5 mg/L, 10 mg/L, 15 mg/L, 20 mg/L, 25 mg/L) were prepared, and the absorbance of the corresponding organic pollutant solutions at the maximum absorption wavelength was determined at different concentrations, and the linear relationship between the organic pollutant absorbance and the organic pollutant concentration was fitted by the organic pollutant absorbance and the organic pollutant concentration, and the superiority or inferiority of the fitting relationship was determined by the correlation coefficient, R^2. The correlation coefficient R^2 was used to determine the superiority of the fitting relationship (control above 0.99). As a result, standard curves for the organic pollutant solutions were obtained and the relevant parameters of the fitted standard curve equations are shown in Fig. S3 below. (The standard curve equation: $y = 0.0353\,x + 0.0643$, $R^2 = 0.992$)

The adsorption procedure of methylene blue and crystal violet is similar to the preparation of malachite green.

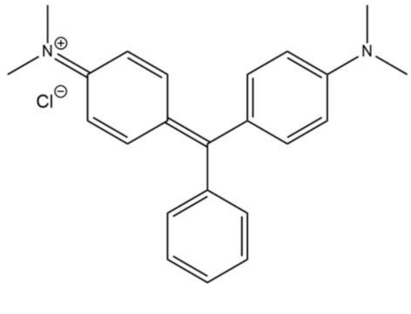

Figure. S2 Structure of the molecular formula of malachite green

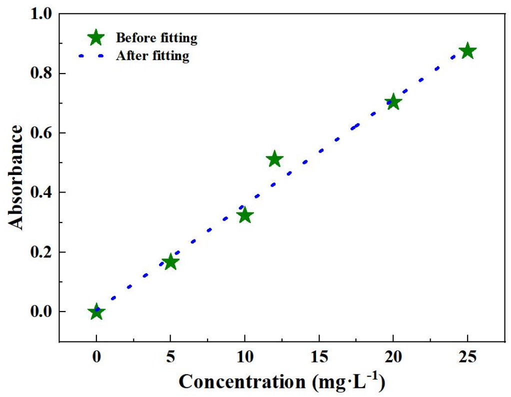

Figure. S3 Standard curve of absorbance values for different concentrations of MG dyes

$$Q_t = (C_0 - C_t)V/m \tag{S1}$$

$$C\% = \frac{(C_0 - C_t)}{C_0} \times 100 \tag{S2}$$

Where, Q_t, C -- dye adsorption amount, mg/g; % of dye removed, C%;

C_0, C_t -- initial concentration of organic dyes, concentration of dyes after adsorption, mg/L;

V -- volume of dye added to adsorbent, L; m -- mass of adsorbent, g.
